# Influencing factors and predictive model of live birth involving low-grade blastocyst frozen–thawed transfer: a retrospective study

**DOI:** 10.1186/s40001-023-01045-2

**Published:** 2023-03-13

**Authors:** Yuan Fang, Ye He, Wanlu Wang, Zhiguo Zhang, Ping Zhou, Yunxia Cao, Xiaojin He, Yuping Xu, Zhaolian Wei

**Affiliations:** 1 Department of Obstetrics and Gynecology, The First Affiliated Hospital of Anhui Medical University, No. 218 Jixi Road, Hefei, 230022 Anhui China; 2 NHC Key Laboratory of Study on Abnormal Gametes and Reproductive Tract (Anhui Medical University), No. 81 Meishan Road, Hefei, 230032 Anhui China; 3 Key Laboratory of Population Health Across Life Cycle (Anhui Medical University), Ministry of Education of the People’s Republic of China, No. 81 Meishan Road, Hefei, 230032 Anhui China; 4 Anhui Province Key Laboratory of Reproductive Health and Genetics, No. 81 Meishan Road, Hefei, 230032 Anhui China; 5 Anhui Provincial Engineering Research Center of Biopreservation and Artificial Organs, No. 81 Meishan Road, Hefei, 230032 Anhui China; 6 Anhui Provincial Institute of Translational Medicine, No. 81 Meishan Road, Hefei, 230032 Anhui China

**Keywords:** Low-grade blastocyst, Clinical pregnancy, Predictive model

## Abstract

**Background:**

Whether only low-grade blastocysts should undergo freeze–thaw transfer during the in vitro fertilization/intracytoplasmic sperm injection cycle remains controversial; however, high-quality embryos cannot be obtained from some patients. Therefore, we aimed to identify factors that may affect the live birth.

**Methods:**

A total of 662 couples with only low-grade blastocysts who voluntarily accepted freeze–thaw blastocyst transfer at a single reproductive center over a 7-year period were followed-up. According to the outcome after transfer, they were divided into live birth group and failed pregnancy group. A nomogram was constructed for predicting live births.

**Results:**

Baseline information and clinical treatment characteristics of patients in the two groups were comparable. Fifty-two of the 662 cycles (7.9%) resulted in live birth. Paternal age, maternal basal luteinizing hormone level, endometrial preparation scheme, and blastocyst development days were independent factors that affected low-grade blastocyst freeze–thaw transfer outcomes. The predictive model constructed based on these four factors presented favorable calibration and discriminatory abilities (area under the curve, 0.734; 95% confidence interval, 0.781–0.813).

**Conclusions:**

For patients who exclusively underwent low-grade blastocyst freeze–thaw transfer, advanced paternal age and a high level of maternal basal luteinizing hormone adversely affected low-grade blastocyst freeze–thaw transfer outcomes. Artificial cycle preparation of the endometrium and day 5 blastocyst selection may improve the probability of live birth.

## Background

Infertility is defined as no clinical pregnancy after 12 months of unprotected sexual intercourse. In the recent reports, infertility affected 8–12% of couples of childbearing age worldwide [1]. In vitro fertilization (IVF) embryo transfer is a common method of treating infertility, and the clinical pregnancy rate with IVF has reached 55–60% [2]. In addition to age and other infertility factors, the quality of embryos used for transfer is key to successful IVF treatment [3] and the predominant predictor of live birth after transfer.

Despite improvements in embryo culture methods, certain insurmountable challenges regarding the quality of available embryos persist. Embryo quality depends on various factors; however, irreversible factors, such as the advanced age of the couple, decreased reproductive organ function, and poor response to ovarian stimulation, among others, have resulted in the acquisition and transfer of poor-quality embryos in certain couples. It is already clear that poor-quality embryos are associated with lower clinical pregnancy rates and poorer pregnancy outcomes than high-quality embryos, whether at the cleavage stage or the blastocyst stage [4, 5]. Nonetheless, considering the significantly limited quality of embryos available, clinical practitioners and their patients continuously encounter selection difficulties. Whether low-grade blastocysts (LGBs) or embryos should be transferred to patients is controversial. This decision requires the consideration of several factors, including the potential of an LGB leading to live birth, severe complications during clinical pregnancy, and the psychological burden on patients receiving poor-quality blastocyst transfer.

High-quality single-embryo transfer has always been considered the optimal scheme for achieving favorable pregnancy outcomes of IVF embryo transfer [6], whereas LGBs have been associated with lower implantation, clinical pregnancy, and live birth rates than high-quality embryos (clinical pregnancy rate, 41.5–19.2%; live birth rate, 32.3–15.5%) [7]. However, studies of the co-transfer of poor-quality embryos with high-quality embryos resulted in inconsistent conclusions [8, 9]. The transfer of high-quality blastocysts plus one low-quality blastocyst has been reported to produce decreasing clinical pregnancy and live birth rates compared with high-quality single-blastocyst transfer, whether using fresh or frozen embryos [10]. Hill et al. [11] have found that the additional transfer of a lower-quality embryo does not have a detrimental effect on a good-quality blastocyst and results in a small increase in live births. Moreover, a recent review concluded that LGBs have the potential to provide patients with a chance of pregnancy when other options may not be practical or economically feasible. Furthermore, LGBs do not adversely affect pregnancy or perinatal outcomes [12].

In clinic practice, the necessity for LGB transfer has been controversial, and most LGBs have been discarded; however, high-quality embryos cannot be obtained from some patients. To our knowledge, no study has focused on patients who underwent embryo transfer with only LGBs. Therefore, this study aimed to determine the independent factors influencing the live birth rate of LGB frozen–thawed transfer by analyzing the relevant factors involved in IVF/intracytoplasmic sperm injection embryo transfer and construct a predictive model that provides beneficial information regarding clinical treatment to improve the confidence of affected patients.

## Methods

### Study population and design

We retrospectively screened 735 LGBs (score < 3 BB) [12, 13] transfer cycles at the reproductive center of the First Affiliated Hospital of Anhui Medical University between March 2014 and March 2021. Inclusion criteria were as follows: cycles involving frozen–thawed blastocyst transfer, availability of only LGBs, and three or fewer transplanted blastocysts. Only the first transplant cycle per couple was included in the study. Exclusion criteria were as follows: women of advanced age (older than 40 years) and patients with a definite diagnosis of monogenic disease, reproductive tract malformation, or other diseases known to cause implant failure and spontaneous abortion. Cycles of preimplantation, genetic diagnoses, and preimplantation genetic testing were not included in this study, excluding 56 cycles of fresh embryo transfer and 17 cycles of nonautonomous oocytes or sperm. A total of 662 cycles met the eligibility criteria.

### Cryopreservation cycles

The entire cohort of embryos was cryopreserved on day 5 (D5) or day 6 (D6) and vitrified using an open system that allows for direct contact with liquid nitrogen and the embryo, which results in a high cooling rate. At our center, we use the Gardner blastocyst score [14] to evaluate embryo quality. Blastocysts were assigned a numerical score ranging from 1 to 6 based on their degree of expansion and hatching status. The scores were as follows: 1, an early blastocyst with a blastocoel less than half the volume of the embryo; 2, a blastocyst with a blastocoel half the volume or greater than half the volume of the embryo; 3, a full blastocyst with a blastocoel completely filling the embryo; 4, an expanded blastocyst with a blastocoel volume larger than that of the early embryo and a thinning zona; 5, a hatching blastocyst with the trophectoderm starting to herniate through the zona; and 6, a hatched blastocyst with the blastocyst completely escaped from the zona. For blastocysts graded as 3–6 (i.e., full blastocysts onward), the development of the inner cell mass was assessed as follows: A, many tightly packed cells; B, several loosely grouped cells; and C, very few cells. The trophectoderm was assessed as follows: A, many cells forming a cohesive epithelium; B, few cells forming a loose epithelium; and C, very few large cells. Using this scoring system, patients who received low-quality embryos (score < 3 BB) were identified during this study, including all blastocysts scored 3BC, 3CB, 3CC, or 1 and 2.

Regarding natural frozen–thawed embryo transfer cycles, the development of the dominant follicle and endometrium was monitored from day 10 using regular transvaginal ultrasound, urine luteinizing hormone (LH) tests, and serum LH, estradiol, and progesterone levels until ovulation. To prevent luteal phase defects, luteal phase support was provided by dydrogesterone tablets from the day of ovulation (10 mg, three times daily; Duphaston; Abbott Laboratories, Chicago, IL, USA). Regarding artificial frozen–thawed embryo transfer cycles, patients received estradiol valerate (2 mg; Progynova; Bayer, Leverkusen, Germany) from day 3, three times daily, to prepare the endometrium for embryo transfer. Endometrial thickness was monitored using transvaginal ultrasound from day 10; when the endometrium reached or exceeded 8 mm, progesterone was provided (60 mg intramuscular, once daily). On embryo transfer day, required endometrial patterns were type B, type C, or type B–C according to the classification of Gonen and Casper [15]. Type B was defined as an intermediate, isoechogenic pattern characterized by the same reflectivity as that of the surrounding myometrium, with a nonprominent or absent central echogenic line. Type C was defined as a multilayer, triple-line endometrium comprising the prominent outer and central hyperechogenic lines and inner hypoechogenic or black regions.

### Outcome measures and statistical analysis

The serum human chorionic gonadotropin level was measured 14 days after transfer. When serum β-human chorionic gonadotropin (hCG) exceeded 20 IU/L, the diagnosis was hCG positive and luteal support therapy was continued. Transvaginal ultrasonography was performed on day 35 after transfer. Clinical pregnancy was diagnosed based on the presence of an intrauterine pregnancy sac, fetal pole, and original cardiac beat. Positive fetal cardiac activity at 12 weeks of gestation, defined as a persistent pregnancy, and live birth after 28 weeks of gestation were the primary outcomes of this study. Patients with live births were assigned to the live birth group (Group 1), and other patients were assigned to the failed pregnancy group (Group 2).

SPSS (22.0; IBM Corp., Armonk, NY, USA) and R (version 4.0.3) software was used for statistical analyses. Continuous data are presented as mean ± standard deviation. Student’s *t* test was used for normally distributed continuous variables, and the Mann–Whitney *U* test was used for non-normally distributed continuous variables. Categorical variables are expressed as numbers or percentages and were assessed using the Chi-square test. Variables with significant differences (P < 0.25) according to univariate analysis were included in the multivariate logistic regression analysis. Independent factors influencing LGB frozen–thawed transfer outcomes were screened out and a nomogram was established. The predictive ability of the nomogram was evaluated using the receiver-operating characteristic (ROC) curve and area under the ROC curve (AUC). The higher the AUC value (i.e., 0.5–1), the higher the resolution of the nomogram. Furthermore, the calibration curve of the nomogram was used to evaluate the predictive accuracy. Finally, we used a decision curve analysis and clinical impact curve to evaluate the net benefit and clinical applicability of the model. A P value < 0.05 was considered statistically significant in all tests.

## Results

According to the B-ultrasound results, 52 and 610 cycles were assigned to the live birth and nonpregnancy groups, respectively. Basic clinical characteristics of the patients are shown in Table 1. Maternal age, infertility type, infertility duration, number of blastocysts transferred, endometrial thickness, and basal estradiol, LH, and progesterone levels on the trigger day were similarly distributed between groups. The maternal body mass index (BMI) and basal follicle-stimulating hormone levels of Group 1 were significantly lower than those of Group 2 (P < 0.05); however, the estradiol level on the trigger day was higher than that of Group 2 (P < 0.05). Single-factor results revealed that the live birth group was more inclined to using artificial cycles to prepare the endometrium, and the live birth rate of blastocysts on day 5 was higher.

**Table 1:** Demographic and treatment characteristics of patients

Characteristics	Group 1 clinical pregnancy (n = 52)	Group 2 failed pregnancy group (n = 610)	*P* value
Maternal age (years ± SD)	30.60 ± 3.49	31.52 ± 4.48	0.257
Paternal age (years ± SD)	31.00 ± 3.77	33.00 ± 5.62	0.057
Maternal BMI (kg/m^2^)	22.00 ± 3.37	23.21 ± 9.60	0.028
Infertility duration (years)	3.63 ± 2.63	4.19 ± 3.02	0.358
Infertility cause, n (%)			0.461
Female	31 (59.62)	397 (65.08)	
Male	5 (9.61)	75 (12.30)	
Mixed	14 (26.92)	128 (20.98)	
Unexplained	2 (3.85)	10 (1.64)	
Type of infertility, n (%)			0.419
Primary	27 (51.92)	352 (57.70)	
Secondary	25 (48.08)	258 (42.30)	
Gravidity, n (%)			0.173
0	21 (40.38)	332 (54.43)	
1	15 (28.85)	146 (23.93)	
> 1	16 (30.77)	132 (21.64)	
Parity, n (%)			0.234
0	39 (75.00)	478 (78.36)	
1	12 (23.08)	130 (21.31)	
> 1	1 (1.92)	2 (0.33)	
ET cycles			0.137
1	25 (48.08)	366 (60.00)	
2	16 (30.77)	167 (27.38)	
> 2	11 (21.15)	77(12.62)	
Procedure			0.238
IVF	37 (71.15)	384 (62.95)	
ICSI	15 (28.85)	226 (37.05)	
Basal FSH (IU/L)	7.09 ± 2.77	8.33 ± 6.91	0.029
Basal LH (IU/L)	4.78 ± 3.57	8.33 ± 6.92	0.168
Basal E2 (pmol/L)	172.79 ± 111.00	179.00 ± 172.91	0.722
E2 on hCG (pmol/L)	12,252.98 ± 5863.05	10,587.24 ± 5950.73	0.038
LH on hCG (IU/L)	2.86 ± 3.18	3.51 ± 5.77	0.290
P on hCG (nmol/L)	5.29 ± 4.00	4.74 ± 4.55	0.263
Retrieved oocytes	11.77 ± 7.38	10.05 ± 6.54	0.054
Endometrial preparation			0.008
Natural cycles	8 (15.38)	202 (33.11)	
Artificial cycle	44 (84.62)	408 (66.89)	
Endometrial thickness (mm)	10.56 ± 1.59	10.70 ± 1.80	0.986
Number of blastocysts, n (%)			0.913
1	9 (17.31)	102 (16.72)	
≥ 2	43 (82.69)	508 (83.27)	
Blastocyst day, n (%)			0.00
D5	14 (26.92)	56 (9.18)	
D6	38 (73.08)	554 (90.82)	
Blastocyst score, n (%)			0.712
3BC or 3CB	36 (69.23)	451 (73.93)	
3CC	14 (26.92)	134 (21.97)	
< 3CC	2 (3.85)	25 (4.1)	

BMI, body mass index; D5, day 5; D6, day 6; E2, estradiol; ET, embryo transfer; FSH, follicle-stimulating hormone; hCG, human chorionic gonadotropin; ICSI, intracytoplasmic sperm injection; IVF, in vitro fertilization; LH, luteinizing hormone

To determine the effect of treatment methods on outcomes, based on the results of single-factor analysis, we divided all cycles into two groups according to different endometrial preparation methods (Table 2). The two groups showed similar performance in terms of maternal age, years of infertility, type of infertility, endometrial thickness before transfer, and probability of three-line characteristics of endometrium, which proved that our treatment plan for them was effective. Simultaneously, it was observed that patients with higher maternal BMI preferred the artificial cycle protocol to prepare the endometrium (P < 0.05), which appeared to complete the endometrium preparation faster than the natural cycle (P < 0.05). The positive hCG, clinical pregnancy, ongoing pregnancy, and live birth rates in the artificial cycle group were significantly higher than those in the natural cycle group.

**Table 2:** Clinical features and outcomes of two endometrial preparation protocols

	Artificial cycle (n = 452)	Natural cycle (n = 210)	*P* value
Maternal age (years)	31.26 ± 4.50	31.61 ± 4.09	0.259
Maternal BMI (kg/m^2^)	22.95 ± 3.34	22.11 ± 2.90	0.003
Infertility duration (years)	4.10 ± 2.90	4.37 ± 3.23	0.496
Type of infertility, *n* (%)			0.836
Primary	260	119	
Secondary	192	91	
Endometrial thickness (mm)	10.48 ± 1.68	10.54 ± 1.48	0.312
Duration of endometrial preparation (days)	12.21 ± 1.08	12.57 ± 1.43	0.006
Triple line endometrium, *n* (%)	388 (85.84)	171 (81.43)	0.145
HCG positive, *n* (%)	52 (11.50)	11 (5.24)	0.006
Clinical pregnancy, *n* (%)	46 (10.18)	9 (4.29)	0.010
Ongoing pregnancy, *n* (%)	44 (9.73)	8 (3.81)	0.008
Live birth, *n* (%)	44 (9.73)	8 (3.81)	0.008

The 662 cycles were then grouped according to the transfer of blastocysts from different developmental days (D5 or D6). Patient characteristics and outcomes are shown in Table 3. Maternal age, paternal age, maternal BMI, years of infertility, type of infertility, and pretransfer endometrial thickness were similarly distributed between the two groups. Although in the D6 blastocyst group, the number of cycles to select multiple blastocysts (≥ 2) for transfer was significantly more than that in the D5 blastocyst group, the positive hCG, clinical pregnancy, continuous pregnancy, and live birth rates in the D5 blastocyst group were significantly higher than those in the D6 blastocyst group.

**Table 3:** Patient characteristics and outcomes of transferred D5 or D6 blastocysts

	D5 blastocyst (n = 70)	D6 blastocyst (n = 592)	*P* value
Maternal age (years)	32.21 ± 4.71	31.36 ± 4.38	0.165
Paternal age (years)	33.81 ± 6.87	33.00 ± 5.33	0.529
Maternal BMI (kg/m^2^)	22.65 ± 3.57	22.69 ± 3.19	0.961
Infertility duration (years)	4.10 ± 3.04	4.15 ± 3.00	0.843
Type of infertility, n (%)			0.569
Primary	38 (54.29)	341 (57.60)	
Secondary	32 (45.71)	251 (42.40)	
Endometrial thickness (mm)	10.53 ± 1.90	10.73 ± 1.76	0.293
Number of blastocysts, n (%)			0.000
1	15 (21.43)	96 (16.22)	
≥ 2	55 (78.57)	496 (83.78)	
HCG positive, n (%)	19 (27.14)	44 (7.43)	0.000
Clinical pregnancy, n (%)	15 (21.43)	40 (6.76)	0.000
Ongoing pregnancy, n (%)	14 (20.00)	38 (6.42)	0.000
Live birth, n (%)	14 (20.00)	38 (6.42)	0.000

**Fig. 1:**
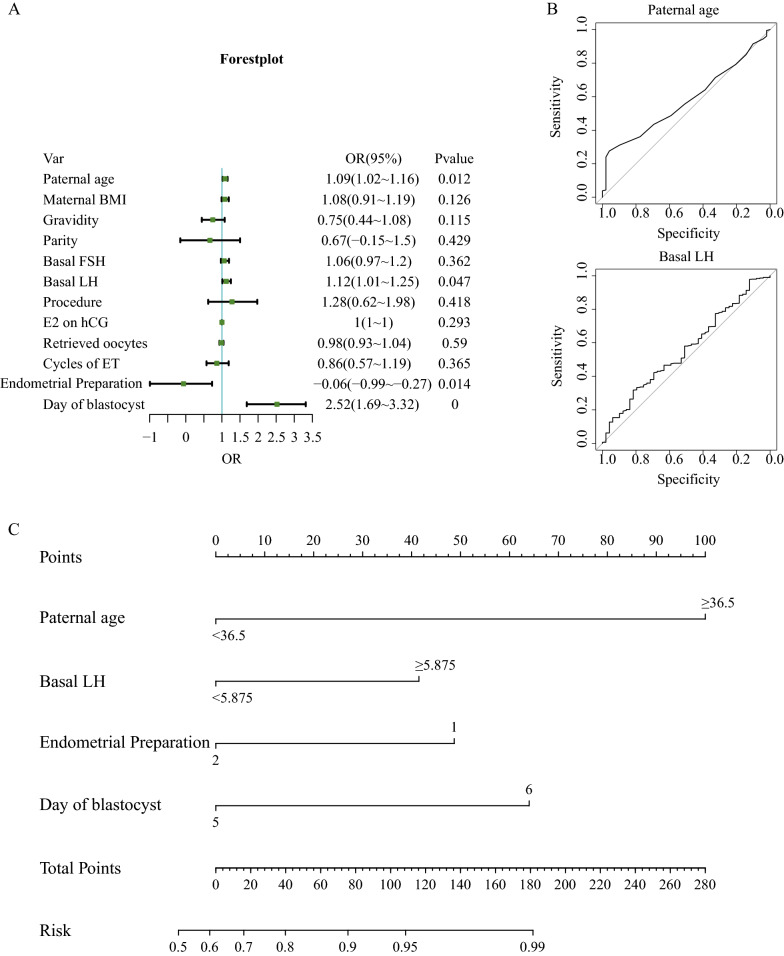
Construction of the nomogram. **A** Forest plot of the multivariate logistic regression analysis results. **B** ROC curves for paternal age and basal LH. **C** Nomogram for predicting clinical pregnancy with low-grade blastocyst frozen–thawed transfer. BMI, body mass index; **e2** estradiol; ET, embryo transfer; FSH, follicle-stimulating hormone; hCG, human chorionic gonadotropin; LH, luteinizing hormone; OR, odds ratio; ROC, receiver operating characteristic

To control bias resulting from confounding factors, factors with differences according to the univariate analysis (P < 0.25) were included in the multivariate logistic regression model. Figure 1A shows the results of multivariate regression using a forest diagram; paternal age (odds ratio [OR], 1.09; 95% confidence interval [CI] 1.02–1.16; *P* = 0.012), basal LH (OR, 1.12; 95% CI 1.01–1.25; *P* = 0.047), endometrial preparation protocol (OR, − 2.06; 95% CI − 2.99 to -1.27; *P* = 0.014), and days of blastocyst development (OR, 2.52; 95% CI 1.69–3.32; *P* = 0.000) were independent factors that influenced LGB frozen–thawed transfer. To ensure the objectivity of the model, we constructed ROC curves (Fig. 1B) for paternal age and basal LH to determine the optimal cutoff value. Using multivariate analysis results and paternal age, basal LH, endometrial preparation protocol, and blastocyst development days as influencing factors, we constructed a nomogram (Fig. 1C) in which paternal age was the greatest contributor, followed by blastocyst development days, endometrial preparation, and basal LH levels. Based on the weighting indexes of multivariate logistic regression analysis, we derived the following formula: Y = 2.338 × paternal age + 1.011 × basal LH − 1.094 × endometrial preparation + 1.552 × day of blastocyst − 7.559.

**Fig. 2:**
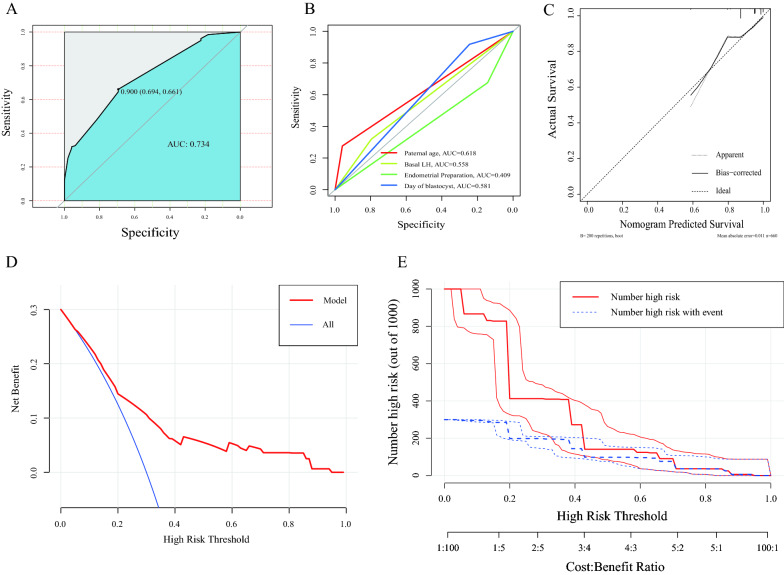
Verification of the nomogram. **A** ROC curve of the nomogram. The AUC is 0.734 (95% confidence interval, 0.800–0.838). **B** ROC curve for each factor in the nomogram: paternal age (AUC, 0.618), basal LH (AUC, 0.558), endometrial preparation (AUC, 0.409), and days of the blastocyst (AUC, 0.581). **C** Calibration plots of the nomogram. **D** Decision curve analysis of the nomogram. **E** Clinical impact curve of the nomogram. AUC, area under the curve; LH, luteinizing hormone; ROC, receiver-operating characteristic

To test the reliability of the model, we constructed a ROC curve for the predictive model (Fig. 2A) with an AUC value of 0.734 (95% CI 0.781–0.813), demonstrating that the current model exhibited favorable predictive power and was superior to paternal age (AUC, 0.618), basal LH (AUC, 0.558), endometrial preparation method (AUC, 0.409), and days of blastocyst development (AUC, 0.581) as compared to single-factor ROC curves (Fig. 2B). Figure 2C shows the calibration curve of the prediction model. Evidently, the calibration curve of the prediction model was consistent with the observation results. Furthermore, to evaluate the clinical applicability of the predictive nomogram, decision curve (Fig. 2D) and clinical impact curve (Fig. 2E) analyses were simultaneously conducted. They visually demonstrated that our predictive model had obvious net value within a wide range of threshold probability; thus, confirming the favorable clinical application value of the nomogram.

## Discussion

To our knowledge, this is the first study to report the outcomes and influencing factors of patients who undergo only LGB frozen–thawed transfer. Clinical pregnancy was achieved with 55 of 662 cycles (8.3%), and live birth was achieved with 52 cycles (7.9%); these rates were considerably lower than those of high-quality blastocyst transfer. We observed that older paternal age, increased maternal basal LH levels, endometrial preparation protocols, and longer blastocyst development days were significantly associated with poor frozen–thawed LGB transfer outcomes. Furthermore, based on these findings, a live birth model for LGB was constructed for the first time.

In our prediction model, paternal age was the greatest contributing factor affecting transfer outcomes; however, this has been rarely reported. Couples worldwide are having children at older ages, which is associated with fertility challenges. Although maternal age appears to have a greater influence on assisted reproductive technology outcomes, a previous study [16] revealed that in a cohort of 17,000 intrauterine insemination cycles, after controlling for maternal age, male age over 35 years was associated with a 32.4% spontaneous abortion rate; however, male age under 35 years was associated with a 13.7% spontaneous abortion rate. Furthermore, du Fossé et al. [17] have observed that older paternal age was associated with increased early spontaneous abortion rates of normal pregnancies, especially for men 45 years or older. After controlling for maternal age using the donor oocyte model, older male age was found to have a significantly negative impact on pregnancy outcomes and blastocyst formation rates [18]. In addition, a retrospective analysis of frozen–thawed embryo transfer cycles conducted at two assisted reproductive technology centers revealed that, after grouping according to paternal age, clinical pregnancy and live birth rates decreased marginally for men older than 30 years, but that there was no statistical difference [19]. Our results suggest that paternal age has a greater impact on the live birth rate of LGBs than other factors considered during this study; however, the exact reason remains unclear. It is possible that older age tends to lead to a decreased blastocyst formation rate. During our study, women older than 40 years were excluded because of known pregnancy difficulties and serious pregnancy complications associated with older maternal age [20]; however, we did not limit the paternal age.

The day of blastocyst development was a significant factor affecting the live birth rates associated with LGBs in our model. The influence of the embryonic development stage on transfer outcomes has been controversial. With the application of vitrification freezing technology, the selection of frozen embryos has evolved from the earlier cleavage stage to the blastocyst stage. When evaluating multiple randomized, controlled trials, Glujovsky et al. [21] observed that blastocyst stage transfer was associated with higher clinical pregnancy and live birth rates than cleavage stage embryos. However, some researchers believe that blastocyst embryo transfer has no advantages compared with cleavage embryo transfer in terms of sustained pregnancy rates and spontaneous abortion rates [22]. Nevertheless, in both cases, the quality of evidence is low. Therefore, more elaborate randomized, controlled trials are required to prove these assertions. Regarding embryo selection at the blastocyst stage, most studies have revealed [23, 24] that day 5 blastocyst transfer potentially results in higher implantation and clinical pregnancy rates than day 6 blastocyst transfer, which is consistent with our low-grade embryo research results. The live birth rate of day 5 blastocysts is significantly higher than that of day 6 blastocysts, possibly because blastocysts with slow development have a higher aneuploidy rate and day 5 blastocysts have more genetic advantages [25]. Successful embryo implantation depends on favorable endometrial receptivity [26], and the endometrium on day 5 after ovulation is more conducive to day 5 blastocyst implantation. Because of slow development, they continue to be cultured to day 6 blastocysts, which have inferior cell quality compared to day 5 blastocysts.

The preparation method of endometrium is one of the key factors to determine the success of frozen– thawed blastocyst transplantation, including natural and artificial cycles. Several studies [27, 28] have reported that insufficient randomized, controlled trials have provided adequate evidence to ascertain which is the more reliable scheme. Some studies [29, 30] have suggested that the natural cycle is not inferior to the artificial cycle in terms of continued pregnancy rates and seems to have greater advantages such as a lower cost because of the elimination of drug treatment. Although the answer to this question is controversial, anovulatory women may require a programmed artificial cycle, and it is more reasonable for normally ovulating women to accept a natural cycle regimen [31]. The results of our study suggest that artificial cycle preparation of the endometrium may be more favorable for live birth rates associated with LGBs, possibly because most women in our study had difficult spontaneous ovulation or irregular menstrual cycles, and their endocrine disorders were potentially responsible for the acquisition of poorly rated blastocysts.

As a significant physiological regulator in women, LH not only participates in the menstrual cycle but also has a critical role in reproductive function. Previous studies on the effects of basal LH levels of IVF have predominantly focused on populations with polycystic ovary syndrome (PCOS). The generally high LH levels during the follicular phase of patients with PCOS potentially lead to decreased pregnancy continuation rates and early pregnancy losses [32]. A recent study by Singh et al. [33] have demonstrated that higher basal LH levels had no statistically significant effect on embryogenesis and clinical pregnancy rates of women with PCOS who underwent IVF; nonetheless, they were associated with significantly reduced fertilization rates. Regardless of the studies that focused exclusively on women with PCOS, some retrospective analyses suggested that increased follicular-phase LH levels (> 8 IU/L) during IVF embryo transfer potentially led to lower oocyte fertilization [34] and pregnancy [35] rates. During the early follicular stage, the reproductive tract is exposed to high LH concentrations [36], which significantly reduces the probability of pregnancy. These results are consistent with our findings.

This study had some limitations. First, our nomogram is based on the retrospective data of 7-year follow-up of a single center. Although strict screening methods are used to reduce the inherent bias, the decisions of clinicians may also affect our results. In the future, it is necessary to verify the discovery that paternal age is the largest contributor to the nomogram in prospective studies. Second, this study focused exclusively on the transplant outcomes of frozen–thawed blastocysts. Therefore, our analysis may not be applicable to fresh transplanted LGBs. Third, the factors we evaluated were limited. The data regarding certain life factors, such as smoking frequency and alcohol consumption of the couple, were not collected. Therefore, we intend to incorporate these aspects in future studies.

## Conclusion

This single-center retrospective analysis is the first to assess the factors influencing frozen–thawed LGB transfer for patients with only poor-quality embryos and establish a prediction model of live birth. Our results suggest that older paternal age and increased maternal basal LH levels are associated with lower live birth rates. However, the artificial cycle as the preimplantation endometrial preparation approach and day 5 blastocyst transfer may increase live birth rates when conducted using only LGBs.

## Data Availability

The datasets used and/or analyzed during the current study are available from the corresponding author on reasonable request.

## Abbreviations

AUC: Area under the ROC curve
BMI: Body mass index
IVF: In vitro fertilization
LGBs: Low-grade blastocysts
hCG: Human chorionic gonadotropin
LH: Luteinizing hormone
OR: Odds ratio
PCOS: Polycystic ovary syndrome
ROC: Receiver-operating characteristic
